# Activation of Epidermal Growth Factor Receptor Sensitizes Glioblastoma Cells to Hypoxia-Induced Cell Death

**DOI:** 10.3390/cancers12082144

**Published:** 2020-08-03

**Authors:** Anna-Luisa Luger, Nadja I. Lorenz, Hans Urban, Iris Divé, Anna L. Engel, Florian Strassheimer, Katja Dettmer, Pia S. Zeiner, Shabnam Shaid, Nina Struve, Malte Kriegs, Ute Hofmann, Peter J. Oefner, Patrick N. Harter, Joachim P. Steinbach, Michael W. Ronellenfitsch

**Affiliations:** 1 Dr. Senckenberg Institute of Neurooncology, University Hospital Frankfurt, Goethe University, 60528 Frankfurt am Main, Germany; Anna-Luisa.Luger@kgu.de (A.-L.L.); nadja.lorenz@googlemail.com (N.I.L.); Hans.Urban@kgu.de (H.U.); iris.dive@kgu.de (I.D.); anna.engel@outlook.com (A.L.E.); strassheimer@med.uni-frankfurt.de (F.S.); Pia.Zeiner@kgu.de (P.S.Z.); Joachim.Steinbach@kgu.de (J.P.S.); 2 University Cancer Center Frankfurt (UCT), University Hospital Frankfurt, Goethe University, 60590 Frankfurt am Main, Germany; Patrick.Harter@kgu.de; 3 German Cancer Consortium (DKTK), Partner Site Frankfurt/Mainz, 60590 Frankfurt am Main, Germany; 4 Frankfurt Cancer Institute (FCI), University Hospital Frankfurt, Goethe University, 60596 Frankfurt am Main, Germany; 5 Institute of Functional Genomics, University of Regensburg, 93053 Regensburg, Germany; Katja.Dettmer@klinik.uni-regensburg.de (K.D.); Peter.Oefner@klinik.uni-regensburg.de (P.J.O.); 6 Department of Medicine, Hematology/Oncology, University Hospital Frankfurt, Goethe University, 60590 Frankfurt am Main, Germany; Shabnam.Shaid@kgu.de; 7 Laboratory of Radiobiology and Experimental Radiation Oncology, Hubertus Wald Tumorzentrum-University Cancer Center Hamburg, University Medical Center Hamburg-Eppendorf, 20246 Hamburg, Germany; ni.struve@uke.de (N.S.); m.kriegs@uke.de (M.K.); 8 Dr. Margarete Fischer-Bosch Institute of Clinical Pharmacology and University of Tübingen, 70376 Stuttgart, Germany; Ute.Hofmann@ikp-stuttgart.de; 9 Institute of Neurology (Edinger Institute), Goethe University, 60590 Frankfurt, Germany

**Keywords:** glioblastoma, EGFR, EGFR*vIII* mutation, hypoxia, starvation

## Abstract

Background: The epidermal growth factor receptor (EGFR) signaling pathway is genetically activated in approximately 50% of glioblastomas (GBs). Its inhibition has been explored clinically but produced disappointing results, potentially due to metabolic effects that protect GB cells against nutrient deprivation and hypoxia. Here, we hypothesized that EGFR activation could disable metabolic adaptation and define a GB cell population sensitive to starvation. Methods: Using genetically engineered GB cells to model different types of EGFR activation, we analyzed changes in metabolism and cell survival under conditions of the tumor microenvironment. Results: We found that expression of mutant EGFR*vIII* as well as EGF stimulation of EGFR-overexpressing cells impaired physiological adaptation to starvation and rendered cells sensitive to hypoxia-induced cell death. This was preceded by adenosine triphosphate (ATP) depletion and an increase in glycolysis. Furthermore, EGFR*vIII* mutant cells had higher levels of mitochondrial superoxides potentially due to decreased metabolic flux into the serine synthesis pathway which was associated with a decrease in the NADPH/NADP+ ratio. Conclusions: The finding that EGFR activation renders GB cells susceptible to starvation could help to identify a subgroup of patients more likely to benefit from starvation-inducing therapies.

## 1. Introduction

Glioblastoma (GB) is the most common primary malignant brain tumor in adults [1]. The current first line standard of care includes surgery followed by radiochemotherapy with temozolomide [2]. This multimodal treatment yields a median overall survival of approximately 15 months [2]. Recently, the addition of tumor-treating fields was shown to prolong overall survival [3]. Nevertheless, tumor recurrence is almost always inevitable and, to this day, no standardsecond or third line treatment for GBs has been established.

The most frequent genetically altered and activated signaling cascade in GBs is the receptor tyrosine kinase-phosphatidylinositol 3 (PI 3) kinase-AKT signaling network [4]. Amplifications of the epidermal growth factor receptor (EGFR) gene can be found in up to 50% of GB [5,6,7]. Approximately 50% of EGFR-amplified GBs additionally harbor an activating mutation termed EGFR*vIII* (or EGFR delta), which is defined by deletion of exons 2–7 and results in ligand-independent signaling [8]. Mammalian target of rapamycin complex 1 (mTORC1) is a multiprotein complex kinase downstream of EGFR that regulates cell growth, proliferation, and metabolism. In addition to EGFR signal transduction, various other signals converge on mTORC1, including nutrient and oxygen availability [9]. Because of the high frequency of activating mutations in the signaling network, EGFR/EGFR*vIII* and mTORC1 are plausible therapeutic targets. Disappointingly, clinical trials targeting EGFR/EGFR*vIII* or mTORC1 have produced negative results [10,11,12,13,14]. As a potential explanation, we previously showed that EGFR and mTORC1 inhibition can exert detrimental metabolic changes that protect GB cells against nutrient deprivation and hypoxia. Both are central features of the GB microenvironment [15,16]. Conversely, we could also demonstrate that decoupling and unphysiological activation of mTORC1 signaling by gene suppression of the physiological mTORC1 inhibitor tuberous sclerosis complex 2 (TSC2, also known as tuberin) sensitizes GB cells to hypoxia-induced cell death [17]. This effect was accompanied by an array of metabolic changes including increased respiration and induction of enzymes of the pentose phosphate pathway [17]. Rarely occurring in GBs, the clinical implications of TSC mutations might be limited to this small subgroup of tumors. Accounting for the high frequency of activating EGFR mutations in GBs, we here used a genetic model of a constitutively active EGFR*vIII* mutant. We hypothesized that EGFR activation might trigger a phenotype similar to TSC2 gene suppression. Here, we report that activation of EGFR signaling induces metabolic changes including a decrease in NAPDH levels that render GB cells more vulnerable to hypoxia-induced cell death. These results warrant further exploration of antiangiogenic therapies in EGFR-activated GBs.

## 2. Results

### 2.1. EGFRvIII Expression Sensitizes Human GB Cells to Hypoxia-Induced Cell Death

**Figure 1:**
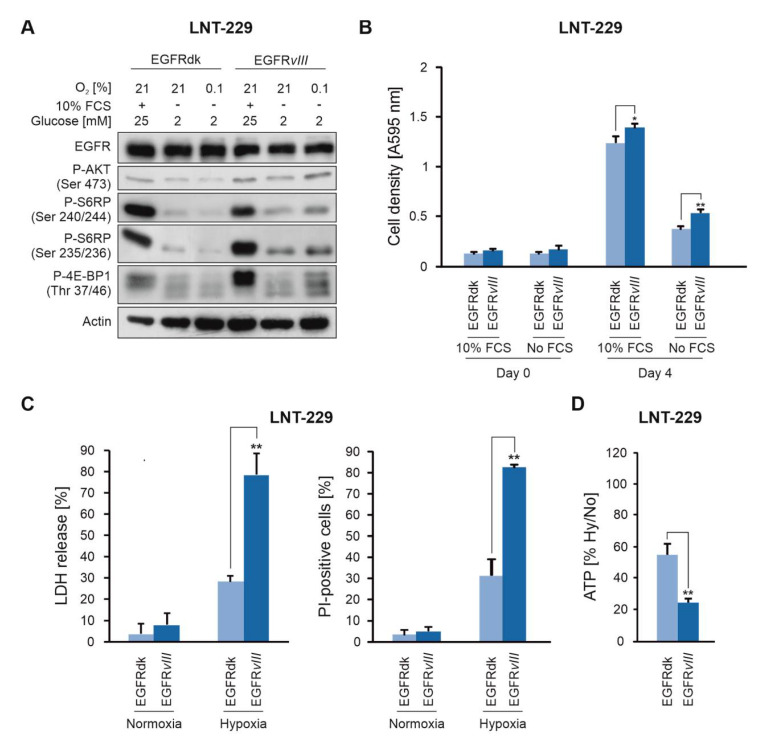
EGFR*vIII* sensitizes human malignant glioma cells to hypoxia-induced cell death. (**A**), LNT-229 EGFRdk and EGFR*vIII* cells were incubated as indicated in medium containing 10% fetal calf serum (FCS) and 25 mM glucose under normoxic conditions, or in serum-free medium containing 2 mM glucose under normoxia (21% oxygen) or hypoxia (0.1% oxygen). Cell lysates were analyzed by immunoblot with antibodies against EGFR, P-Akt (Ser 473), P-S6RP (Ser 240/244 and Ser 235/235), P-4E-BP1 (Thr 37/46) or actin. (**B**), LNT-229 EGFRdk and *vIII* cells were incubated in serum-free and serum containing (10% FCS) culture conditions without glucose restriction (25 mM glucose). Cell density was measured by crystal violet staining (*n* = 4, mean ± S.D., * *p* < 0.05, ** *p* < 0.01). (**C**–**D**), LNT-229 EGFRdk and EGFR*vIII* cells were exposed to glucose restricted (2 mM glucose) serum-free medium under normoxic or hypoxic (0.1% oxygen) conditions. (**C**), Cell death was quantified by LDH-release (*n* = 4, mean ± S.D., ** *p* < 0.01) and propidium iodide staining (*n* = 3, mean ± S.D., ** *p* < 0.01). (**D**), ATP was quantified by a luciferase-based assay. The hypoxia-to-normoxia ratio (Hy/No) of ATP concentrations is shown (*n* = 5, mean ± S.D., ** *p* < 0.01).

We previously showed that inhibition of EGFR and mTORC1 protects glioma cells from hypoxia-induced cell death [15,16]. Furthermore, we recently reported that mTORC1 activation sensitizes to hypoxia-induced cell death and described mTORC1 activation as a metabolically targetable Achilles’ heel in glioma [17]. We hypothesized that EGFR activation, similar to mTORC1 activation, causes metabolic changes that render GB cells vulnerable to nutrient and oxygen deprivation. Since EGFR amplification and mutation are frequently lost in cultured GB cells [18], we used genetic induction of a constitutively active EGFR*vIII* mutant to assess metabolic effects. In an exploratory approach, we could further show that LNT-229 EGFR*vIII* cells also display an increased downstream signaling under starvation as well as an enhanced sensitivity to hypoxia-induced cell death [17]. On the basis of our previous results [17], we analyzed phosphorylation of different EGFR and mTORC1 target proteins. In nutrient rich culture medium, no relevant differences in the phosphorylation status were detected under normoxia (Figure 1A). In contrast, under glucose deprivation and serum-free conditions, a marked decrease in phosphorylation of AKT, S6 ribosomal protein (S6RP) and eukaryotic translation initiation factor 4E (eIF4E)-binding protein 1 (4E-BP1) was observed under both normoxia and hypoxia. However, this effect was markedly impaired in cells expressing EGFR*vIII* (Figure 1A). Cell growth was slightly increased in EGFR*vIII* cells compared to control cells (EGFRdk) both in the presence and absence of serum (Figure 1B). EGFR*vIII*-mediated signaling sensitized GB cells to hypoxia-induced cell death (Figure 1C) and ATP levels were correspondingly decreased under hypoxic conditions (Figure 1D).

### 2.2. EGFRvIII Enhances Glucose Consumption and Lactate Production under Hypoxic Conditions and Reduces Flux Into the One-Carbon Metabolism

**Figure 2:**
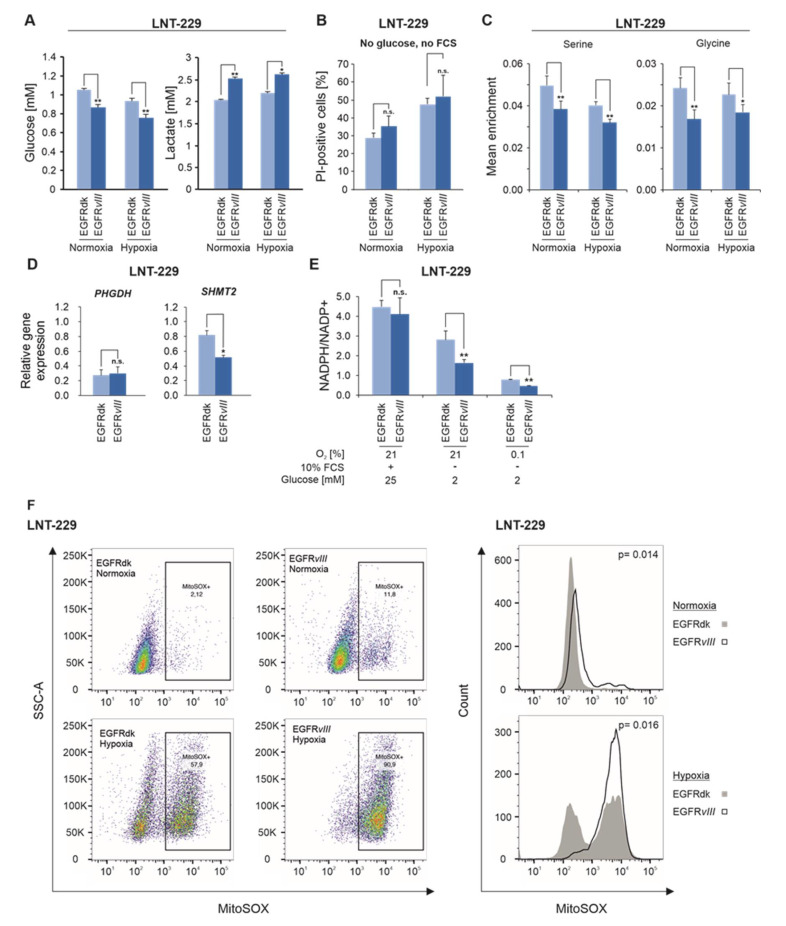
EGFR*vIII* enhances glucose consumption and lactate production under starvation conditions. (**A**) Cells were incubated in glucose restricted (2 mM glucose) serum-free medium under normoxic or hypoxic (0.1% oxygen) conditions. Glucose and lactate concentration were determined in the supernatant (*n* = 3, mean ± S.D., * *p* < 0.05, ** *p* < 0.01). (**B**) LNT-229 EGFRdk and EGFR*vIII* cells were exposed to glucose- and serum-free medium under normoxic or hypoxic (0.1% oxygen) conditions. Cell death was quantified by propidium iodide staining (*n* = 3, mean ± S.D., n.s. = not significant). (**C**) Cells were exposed to glucose restricted (2 mM D-Glucose-^13^C_6_) serum-free medium under normoxic conditions or 0.1% oxygen. Analysis of amino acid isotopologues was performed by HPLC-ESI-MS/MS (*n* = 3, mean ± S.D., * *p* < 0.05, ** *p* < 0.01). (**D**) cDNA of cells was generated and gene expression of *PHGDH* and *SHMT2* was analyzed by qPCR. Values are normalized to *18S* as well as *SDHA* housekeeping gene expression (*n* = 3, mean ± S.D., n.s. = not significant, * *p* < 0.05). (**E**), Cells were incubated as indicated. Analysis of NADPH/NADP^+^ ratios was performed by a luminescence-based assay (*n* = 3, mean ± S.D., n.s. = not significant, ** *p* < 0.01). (**F**), Cells were incubated in serum-free medium with glucose deprivation (2 mM glucose) in normoxia (21% oxygen) or in combination with hypoxia (0.1% oxygen). Analysis of MitoSOX-positive cells was performed by FACS analysis (*n* = 3). The MitoSOX signal of EGFRdk vs. EGFR*vIII* cells is plotted as a function of sideward scatter (SSC-A) (left panel) or cell number (right panel).

EGFR*vIII* cells displayed enhanced glucose consumption and lactate production (Figure 2A), whereas oxygen consumption rates were similar (Figure S1A). This indicates a higher rate of aerobic glycolysis and a less favorable energetic profile in EGFR*vIII* mutant cells. Gene expression of the transcription factors peroxisome proliferator-activated receptor gamma coactivator 1α and -1β, *(PGC-1α* and *PGC-1β)*, which we had previously found induced by proximal mTORC1 activation [17], was unaffected by EGFR*vIII* (Figure S1B). To test whether the energetic efficiency of glucose oxidation is responsible for the observed phenotype, EGFRdk and EGFR*vIII* cells were incubated in the absence of glucose, which resulted in similar proportions of non-viable cells (Figure 2B). To elucidate metabolic alterations in EGFR*vIII* cells, the intracellular flux of glucose-derived carbon atoms was measured via ^13^C-tracing using uniformly ^13^C-labeled glucose. With regard to the one-carbon metabolism, EGFR*vIII* cells displayed a decreased flux of ^13^C-atoms into the serine and glycine pathway under starvation conditions (Figure 2C). Flux of ^13^C-atoms into pyruvate, glutamate, and proline was also reduced significantly, albeit only under normoxia (Figure S2). For all other investigated intermediates of the central carbon metabolism, no significant differences in ^13^C-enrichment were observed (Figure S2). Next, we investigated the mRNA levels of phosphoglycerate-dehydrogenase *(PHGDH)* and serine hydroxymethyltransferase 2 *(SHMT2)*, two central enzymes involved in the synthesis and degradation of serine and glycine. Gene expression of *PHGDH* did not differ between the cell lines (Figure 2D). EGFR*vIII* cells had a lower expression of *SHMT2* (Figure 2D). In line with a reduced shunt in the serine and glycine synthesis pathway, the NADPH/NADP^+^ ratio decreased in EGFR*vIII* cells compared to EGFRdk cells under nutrient starvation and hypoxia (Figure 2E). This effect was accompanied by an increase in superoxide radical production/concentrations under starvation conditions in EGFR*vIII* cells compared to EGFRdk cells (Figure 2F).

Previously, mTORC1 activation was found to increase levels of intermediates of the pentose phosphate pathway [17]. Therefore, a metabolome analysis of EGFR*vIII* and EGFRdk cells was performed. Starvation conditions resulted in reductions in the energy charge and ATP levels in EGFR*vIII* (Figure S3, Figure 1D). No significant difference in intermediates of glycolysis, pentose phosphate pathway and citric acid cycle between EGFRdk and *vIII* cells was detectable (Figure S3).

### 2.3. EGF Enhances the Sensitivity of GB Cells to Hypoxia-Induced Cell Death

**Figure 3:**
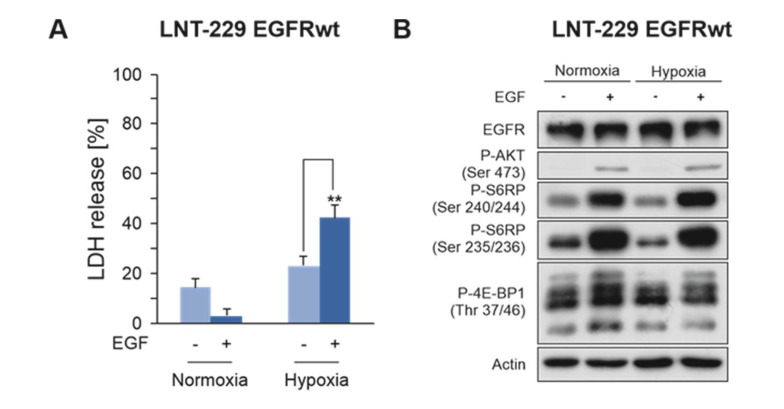
EGF enhances the sensitivity of LNT-229 EGFRwt overexpressing cells to hypoxia-induced cell death. LNT-229 EGFRwt overexpressing cells were exposed to glucose restricted (2 mM glucose) serum-free medium under normoxic or hypoxic (0.1% oxygen) conditions with or without 10 ng/mL EGF. (**A**), Cell death was quantified by LDH-release (*n* = 4, mean ± S.D., ** *p* < 0.01). (**B**), Cell lysates were prepared and analyzed by immunoblot with antibodies for EGFR, P-Akt (Ser 473), P-S6RP (Ser 240/244 and Ser 235/235), P-4E-BP1 (Thr 37/46) or actin.

LNT-229 cells with overexpression of wildtype EGFR (LNT-229 EGFRwt), mimicking the frequently found in vivo situation, are not sensitized to hypoxia-induced cell death in the absence of ligand [17]. However, treatment with epidermal growth factor (EGF) enhanced the sensitivity of EGFR-overexpressing cells to hypoxia-induced cell death (Figure 3A). Compatible with these results, EGF stimulation attenuated inhibition of phosphorylation of AKT, S6RP and 4E-BP1 under starvation conditions (Figure 3B).

### 2.4. EGFR Governs Sensitivity of GB Cells To Hypoxia and Nutrient Deprivation

**Figure 4:**
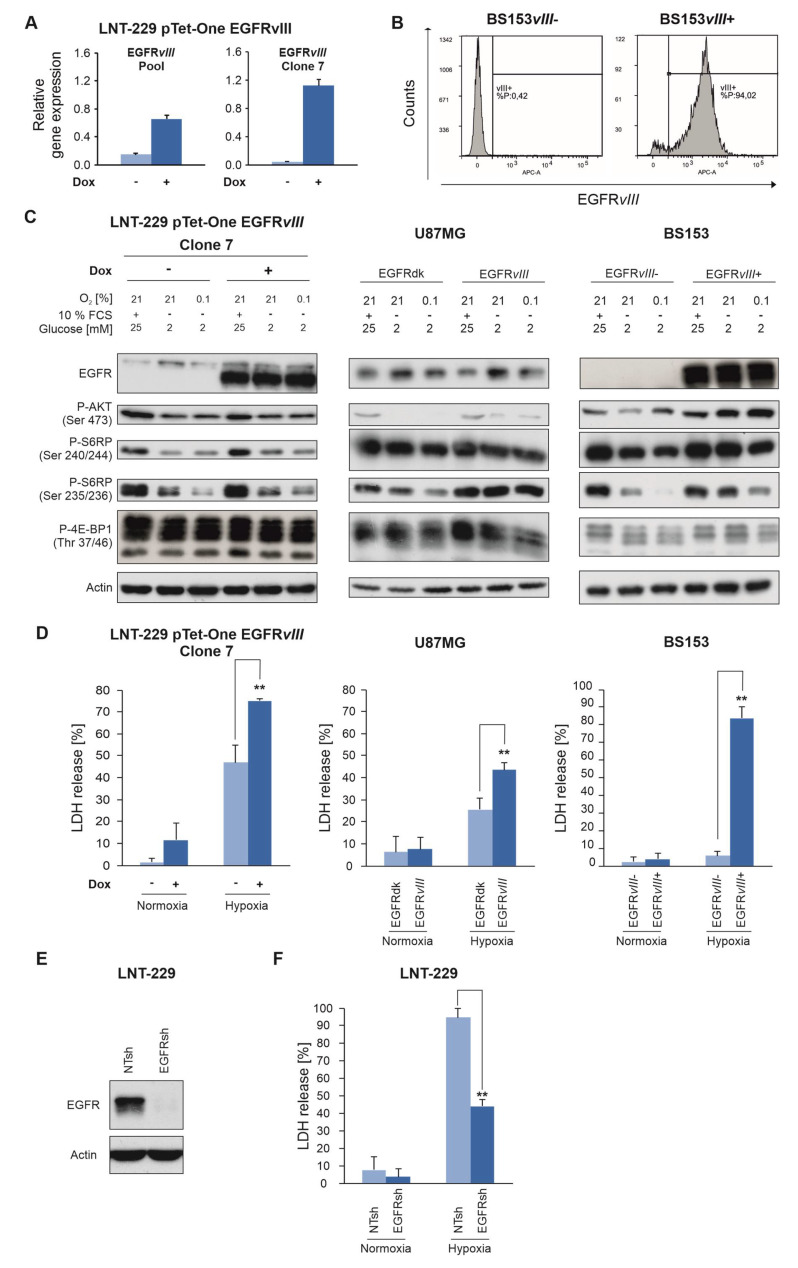
LNT-229 pTet-One EGFR*vIII*, U87MG EGFR*vIII* and BS153 EGFR*vIII*+ cells display enhanced mTORC1 signaling under deprivation conditions and are sensitized to hypoxia-induced cell death. (**A**), Pooled cells and single cell clones of LNT-229 pTet-One EGFR*vIII* cells treated with doxycycline or vehicle were analyzed by qPCR. *EGFRvIII* gene induction by doxycycline was confirmed. Values are normalized to *18S* as well as *SDHA* housekeeping gene expression (*n* = 3, mean ± S.D.). Clone 7 was chosen for further experiments (27-fold increased expression of *EGFRvIII*). (**B**), Using parental BS153 cells, EGFR*vIII*+ and EGFR*vIIII*- sub cell lines were separated by FACS as described [19]. EGFR*vIII* expression in EGFR*vIII*−/+ sub cell lines after sorting was analyzed by FACS. (**C**), LNT-229 pTet-One EGFR*vIII,* U87MG EGFRdk or *vIII* and BS153 EGFR*vIII−* or EGFR*vIII*+ cells were incubated as indicated in medium containing 10% FCS without glucose restriction (25 mM glucose) under normoxic conditions or in FCS-free medium with glucose deprivation (2 mM glucose) under normoxia or in combination with hypoxia (0.1% oxygen). Cell lysates were analyzed by immunoblot with antibodies against EGFR, P-Akt (Ser 473), P-S6RP (Ser 240/244 and Ser 235/235), P-4E-BP1 (Thr 37/46) and actin. (**D**), LNT-229 pTet-One EGFR*vIII*, U87MG EGFRdk or *vIII* and BS153 EGFR*vIII*–or EGFR*vIII*+ cells were exposed to glucose restricted (2 mM glucose) serum-free medium under normoxic or hypoxic (0.1% oxygen) conditions. Cell death was quantified by LDH-release (*n* = 4, mean ± S.D., ** *p* < 0.01). (**E**), LNT-229 NTsh and EGFRsh cells were incubated in serum-free medium without glucose restriction (25 mM glucose) under normoxic conditions. Cellular lysates were analyzed by immunoblot with antibodies for EGFR and actin. (**F**), LNT-229 NTsh and EGFRsh cells were exposed to glucose restricted (2 mM glucose) serum-free medium under normoxic or hypoxic (0.1% oxygen) conditions. Cell death was quantified by LDH-release (*n* = 4, mean ± S.D., ** *p* < 0.01).

To further confirm the robustness of the EGFR*vIII* phenotype, LNT-229 cells that express EGFR*vIII* in a doxycycline-inducible manner were generated (LNT-229 pTet-One EGFR*vIII*) (Figure 4A). Additionally, U87MG EGFRdk or EGFR*vIII* cells as well as BS153 EGFR*vIII*- and EGFR*vIII*+ (Figure 4B) cells were analyzed. Under standard conditions, no difference in the phosphorylation status of downstream target proteins was detectable (Figure 4C). However, incubation of cells under nutrient depleted and hypoxic conditions revealed an increased phosphorylation of S6RP in pTet-One EGFR*vIII* cells compared to control cells without doxycycline treatment (Figure 4C). In U87MG EGFR*vIII* cells as well as BS153 ERGFR*vIII*+ cells, an increased phosphorylation of AKT and S6RP was detectable under these conditions compared to EGFRdk and BS153 EGFR*vIII*− cells (Figure 4C). BS153 EGFR*vIII*− cells shifted the pattern of the 4E-BP1 isoforms toward the faster migrating hypophosphorylated 4EBP1 bands under starvation conditions (Figure 4C). In contrast, BS153 EGFR*vIII*+ cells showed a pattern of more slower-moving hyperphosphorylated bands of 4E-BP1 under starvation conditions, consistent with persistent mTORC1 downstream activity (Figure 4C).

Additionally, LNT-229 pTet-One EGFR*vIII*, U87MG EGFR*vIII* and BS153 EGFR*vIII*+ cells showed an enhanced sensitivity to hypoxia-induced cell death (Figure 4D). We have already shown that pharmacological EGFR inhibition confers protection from hypoxia-induced cell death [16]. Similarly, gene suppression of EGFR (Figure 4E) reversed the phenotype and protected EGFRsh cells from hypoxia-induced cell death (Figure 4F).

### 2.5. The Regional Pattern of EGFRvIII in GBs is Compatible with Increased Vulnerability to Starvation Conditions In Vivo

**Figure 5:**
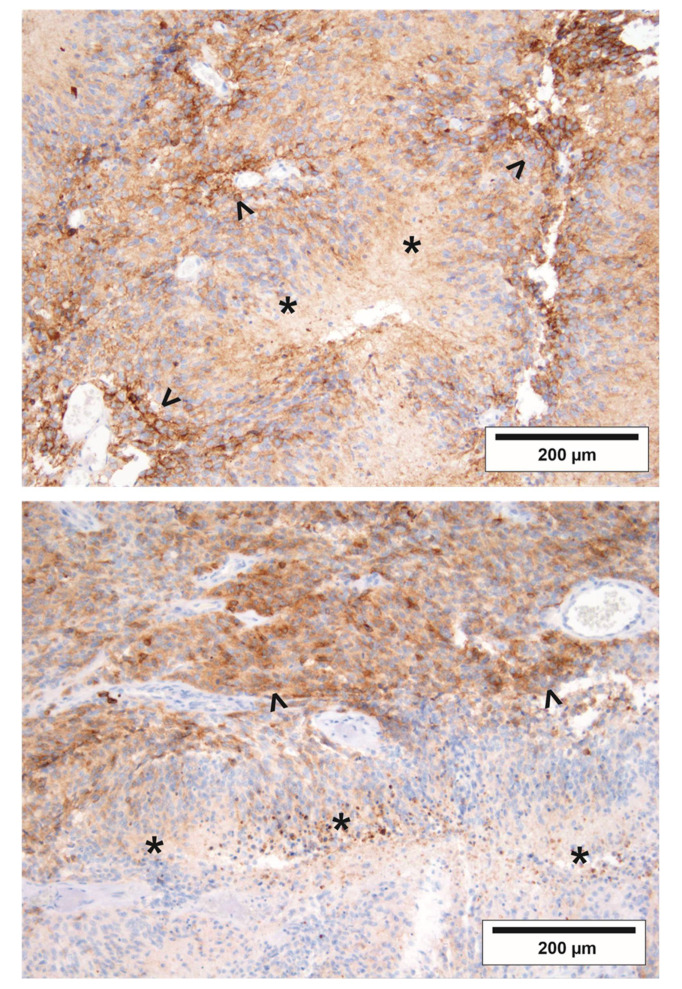
EGFR*vIII* immunohistochemistry of two exemplary GBs. Formalin-fixed, paraffin-embedded tissue of two GBs was analyzed by immunohistochemistry with antibodies for EGFR*vIII*. Asterisks (*) indicate necrosis in GB tissue, arrowheads (>) highlight EGFR*vIII*-positive tumor cells distant from nutrient and oxygen deprived perinecrotic regions.

In EGFR*vIII*-positive GBs, a heterogeneous pattern of EGFR*vIII*-positivity was observed. In line with an enhanced sensitivity to starvation conditions, two exemplary tumors displayed fewer EGFR*vIII*-positive tumor cells in perinecrotic (hypoxic) tumor regions than in regions more distant from the perinecrotic zone (Figure 5).

## 3. Discussion

Given that EGFR signaling is the central genetically activated pathway in GBs, our results reveal a novel, potentially targetable Achilles’ heel in GB subgroups. These results extend our previous finding of enhanced susceptibility of GB cells with impaired mTORC1 regulation to hypoxia-induced cell death [17] to the more prevalent scenario of EGFR activation. Common to both models is a defective inhibition of downstream signaling when nutrients and oxygen become limited (Figure 1A and Figure 4C). With regard to hypoxia, the biological phenotype of constitutive EGFR and mTORC1 activation are similar. Yet the underlying metabolic changes differ (Figures S4 and S5). With an unaffected rate of glucose uptake and increased oxygen consumption, mTORC1 activation by *TSC2* gene suppression reduced aerobic glycolysis, which was enhanced in EGFR*vIII* cells (Figure 2A, Figures S1A and S4). Furthermore, no significant changes in the intracellular levels of metabolites of glycolysis, the citric acid cycle and the pentose phosphate pathway were detected in EGFR*vIII* cells (Figures S2 and S3), while TSC2sh cells showed increased amounts of intermediates of the pentose phosphate pathway (Figure S4) [17], potentially providing NADPH to the detoxification of increased ROS [17]. In EGFR*vIII* cells, an increase in superoxides was detectable (Figure 2F) with a concomitant reduction in the NADPH/NADP^+^ ratio under hypoxia (Figure 2E). The reduced NADPH level could be caused by a reduced glycolytic flux into the serine synthesis pathway (Figure 2C,D) that can generate mitochondrial NADPH via the action of SHMT2. Accordingly, SHMT2 has been reported to be of particular importance under hypoxic conditions [20].

In summary, our results support the notion that activation of EGFR and downstream signaling characterizes a GB subgroup with an enhanced vulnerability to hypoxia-inducing therapies. Antiangiogenic drugs fall into this category and it has been demonstrated that the VEGFA-targeting antibody bevacizumab can cause local therapeutic hypoxia [21,22]. However, bevacizumab has failed to prolong overall survival in unselected GB patients [23]. Likewise, the results of clinical trials exploring EGFR and mTOR inhibition in unselected glioblastoma patients with a one-size-fits-all approach were negative [10,11,12,13,14]. Nonetheless, post-hoc analyses indicated favorable therapeutic effects in a subgroup of tumors with distinct pathway activation [14,24]. Apart from this, the analysis of a patient cohort of one of these large GB trials revealed a positive correlation of phosphorylated S6RP and necrosis, indicating that dysregulation of this cascade could sensitize GB cells to physiological nutrient and/or oxygen deprivation [24]. Based on our findings, these tumors might also be candidates for hypoxia-inducing therapies in future clinical investigations. When moving toward clinical applications, immunohistochemistry of GB tissue samples is a way to probe for EGFR-dysregulation. Including a downstream surrogate marker of pathway activation, such as phosphorylation of S6RP or 4E-BP1, would also cover additional upstream activating events (e.g., PTEN deletion). For a reliable investigation of tissue, a standardized fixation protocol is important because time to tissue fixation or tissue thickness can affect the amount of phosphorylated proteins [25].

To connect our in vitro findings to the results to the aforementioned clinical trials, it would be interesting to investigate EGFR*vIII* GBs in mouse models. Based on our in vitro results, we hypothesize a superior efficacy of starvation or hypoxia-inducing therapies in EGFR*vIII*-mutated vs. wildtype tumors. Additionally, experimental tumors generated by cell mixtures of EGFR-mutated and wildtype cells in the same genetic background (e.g., BS153 EGFR*vIII*− and EGFR*vIII*+) could be used to further investigate distribution patterns of EGFR-activated cells (Figure 5). 

## 4. Materials and Methods

### 4.1. Reagents, Cell Lines and Culture Conditions

All reagents not specified were purchased from Sigma-Aldrich (St. Louis, MO, USA). D-Glucose-^13^C_6_ (99%) was purchased from Euriso-Top (Saint-Aubin, France). LNT-229 cells were maintained as described [15,17]. LNT-229 pLKO.1 and pTetOne-transfected cells were grown in medium containing 2 µg/mL puromycin. pTetOne-transfected cells were treated with doxycycline to induce gene expression of EGFR*vIII*. LNT-229 and U87MG cells transduced with wild-type (pLWERNL), constitutively active (pLERNL EGFR*vIII*), and kinase-deficient (pLERNL EGFRdk) EGFR were cultivated in medium supplemented with 400 µg/mL G418 [17,26]. For comparison of different clones, cell densities were kept equal [15].

### 4.2. Generation of LNT-229 pTetOne EGFRvIII Cells

Human EGFR*vIII* cDNA was amplified from the pLERNL plasmid and cloned into the pTetOne plasmid (Clontech Laboratories, Mountain View, CA, USA) using the cloning sites *Agel-Mlul* (Genscript Biotech, Piscataway Township, NJ, USA). The pTetOne EGFR*vIII* plasmid was transfected into LNT-229 glioma cells using the Xfect transfection protocol (Clontech Laboratories).

### 4.3. Generation of LNT-229 NTsh and EGFRsh Cells

The pLKO.1 plasmids targeting EGFR (EGFRsh) and the pLKO.1 plasmid with a non-targeting shRNA sequence (NTsh) were purchased from Sigma-Aldrich (TRCN0000121068, TRCN0000295969) and Addgene (Watertown, MA, USA, #1864). Lentivirus was produced according to the Addgene protocol in HEK293 cells using the packaging plasmid pCMV-dR8.2 dvpr (Addgene #8455) and the envelope plasmid pCMV-VSVG (Addgene #8454). Polybrene (Millipore, Burlington, MA, USA) was used for transduction.

### 4.4. Generation of BS153 EGFRvIII- and EGFRvIII+ Cells

Flow cytometry was performed as described previously [19] using a FACSCanto (BD Biosciences, San Jose, CA, USA) and Flowlogic software (Miltenyi Biotec, Bergisch Gladbach, Germany). For EGFRvIII detection and quantification anti-EGFRvIII antibody L8A4 (1:1000, mouse, Absolute antibody, #Ab00184-1.4) and Alexa Fluor^TM^ 647 labeled secondary antibody (1:1000, #A-21235, Life Technologies, Carlsbad, CA, USA) were used.

### 4.5. Induction of Hypoxia

Hypoxia was induced with GasPak pouches (Becton Dickinson, Franklin Lakes, NJ, USA) as described [15].

### 4.6. Quantitative Reverse Transcription-PCR (qRT-PCR) Analysis

RNA extraction, cDNA synthesis and qPCR were performed as described previously [17]. Primer pairs are listed in the supplement (Table S1).

### 4.7. Immunoblot Analysis

Immunoblot was performed following a standard protocol [17]. Membranes were probed with antibodies against P-AKT (Ser 473), P-S6RP (Ser 240/244 and Ser 235/235), P-4E-BP1 (Thr 37/46) (Cell Signaling Technology, Danvers, MA, USA), EGFR and actin (Santa Cruz Biotechnology, Dallas, TX, USA). Secondary anti-goat and anti-rabbit antibodies were purchased from Santa Cruz Biotechnology and Jackson ImmunoResearch (West Grove, PA, USA), respectively. Quantification of immunoblot bands was performed by measuring the pixel density of scanned films using ImageJ software (NIH, Bethesda, MD, USA).

### 4.8. Cell Density and Cell Viability Assays

Cell density was assessed by crystal violet (CV) staining as described [17]. Cell viability was inferred from propidium iodide (PI) uptake and lactate dehydrogenase (LDH) release using the Cytotoxicity Detection Kit (LDH) (Roche, Basel, Switzerland) as described previously [17].

### 4.9. Measurement of Glucose, Lactate, ATP and Oxygen

Measurement of glucose and lactate in cell-free supernatant was performed using the biochemistry analyzer Hitachi 917 [17]. ATP levels were analyzed using CLS II kit (Roche) [15,17]. Oxygen consumption was measured with a fluorescence-based assay (PreSens, Regensburg, Germany) [17].

### 4.10. Stable Isotope Tracer Analysis and Quantification of Intracellular Metabolites

A detailed description is included in the supplement (supplementary materials and methods).

### 4.11. NADPH/NADP^+^ Measurement

NADPH and NADP^+^ were measured with a luminescence-based assay (NADP/NADPH-glo assay kit, Promega, Madison, WI, USA) according to the manufacturer’s protocol.

### 4.12. MitoSOX FACS Analysis

MitoSOX was purchased from ThermoFisher (Waltham, MA, USA). Harvested cells were treated with 5 μM MitoSOX in IMDM complete medium for 10 min at 37 °C prior to analysis. Cells were washed twice in phosphate-buffered saline (PBS) followed by the addition of PBS prior to FACS analysis employing a BD FACSCanto II (BD Biosciences) and FlowJo V10 software (Ashland, OR, USA).

### 4.13. Immunohistochemistry of Formalin-Fixed, Paraffin-Embedded Tissue

We performed immunohistochemistry against EGFR*vIII* (mouse anti-human EGFR variant III antibody clone EGFRvIII.1, Zytomed, Berlin, Germany) on two EGFR*vIII* mutant GBs using standard protocols (antibody dilution 1:100, EDTA-buffer antigen retrieval) on a Leica Bond III automated staining system. The use of human GB samples was approved by the ethics committee of Frankfurt University (Ethik-Kommission, University Hospital Frankfurt, Goethe University) with the reference numbers SNO_SNO_01-08.

### 4.14. Statistical Analysis

Quantitative data are expressed as indicated including standard deviation (S.D.). *p*-values were derived from two-tailed student’s t-tests. Values of *p* > 0.05 were considered not significant (n.s.). Values of *p* < 0.05 and *p* < 0.01 were considered significant and highly significant (Excel, Microsoft, Seattle, WA, USA).

## 5. Conclusions

We here identify activation of EGFR signaling as an indicator for susceptibility of GB cells to hypoxia-induced cell death. EGFR-dysregulated cells display defective inhibition of downstream signaling under starvation conditions as well as broad metabolic changes finally leading to energy exhaustion. Given that the signaling cascade governed by EGFR is one of the most frequently genetically activated GB pathways, the study reveals a new, potentially targetable Achilles’ heel and could help to identify subgroups of patients more likely to benefit from starvation-inducing therapies.

## Supplementary Materials

The following are available online at https://www.mdpi.com/2072-6694/12/8/2144/s1. Figure S1: Oxygen consumption and expression of genes of mitochondrial oxidative function are not altered by EGFR*vIII* expression. Figure S2: Intracellular metabolic flux of glucose in LNT-229 EGFRdk and EGFR*vIII* cells measured via ^13^C-flux-analysis. Figure S3: Intracellular metabolic profiles of LNT-229 EGFR*vIII* and dk cells. Figure S4: Schematic overview of the metabolic changes in TSCsh and EGFR*vIII* cells. Figure S5: Schematic model of metabolic changes in EGFRdk and EGFR*vIII* cells. Figure S6: Densitometry readings of immunoblots. Supplementary Materials and Methods: Table S1: Primer pairs for qRT-PCR analysis; Stable isotope tracer analysis; Quantification of intracellular metabolites
