# Supplementary material for: Activation of Epidermal Growth Factor Receptor Sensitizes Glioblastoma Cells to Hypoxia-Induced Cell Death
Source: Cancers (Basel). 2020 Aug 3;12(8):2144. doi: 10.3390/cancers12082144 (PMC7464192; doi:10.3390/cancers12082144)
Supplement: Supplementary file 1 [file cancers-12-02144-s001.pdf]

Supplementary Materials: Activation of Epidermal Growth Factor Receptor Sensitizes Glioblastoma Cells to Hypoxia-Induced Cell Death

Anna-Luisa Luger, Nadja I. Lorenz, Hans Urban, Iris Divé, Anna L. Engel, Florian Strassheimer, Katja Dettmer, Pia S. Zeiner, Shabnam Shaid, Nina Struve, Malte Kriegs, Ute Hofmann, Peter J. Oefner, Patrick N. Harter, Joachim P. Steinbach and Michael W. Ronellenfitsch

Supplementary Figures

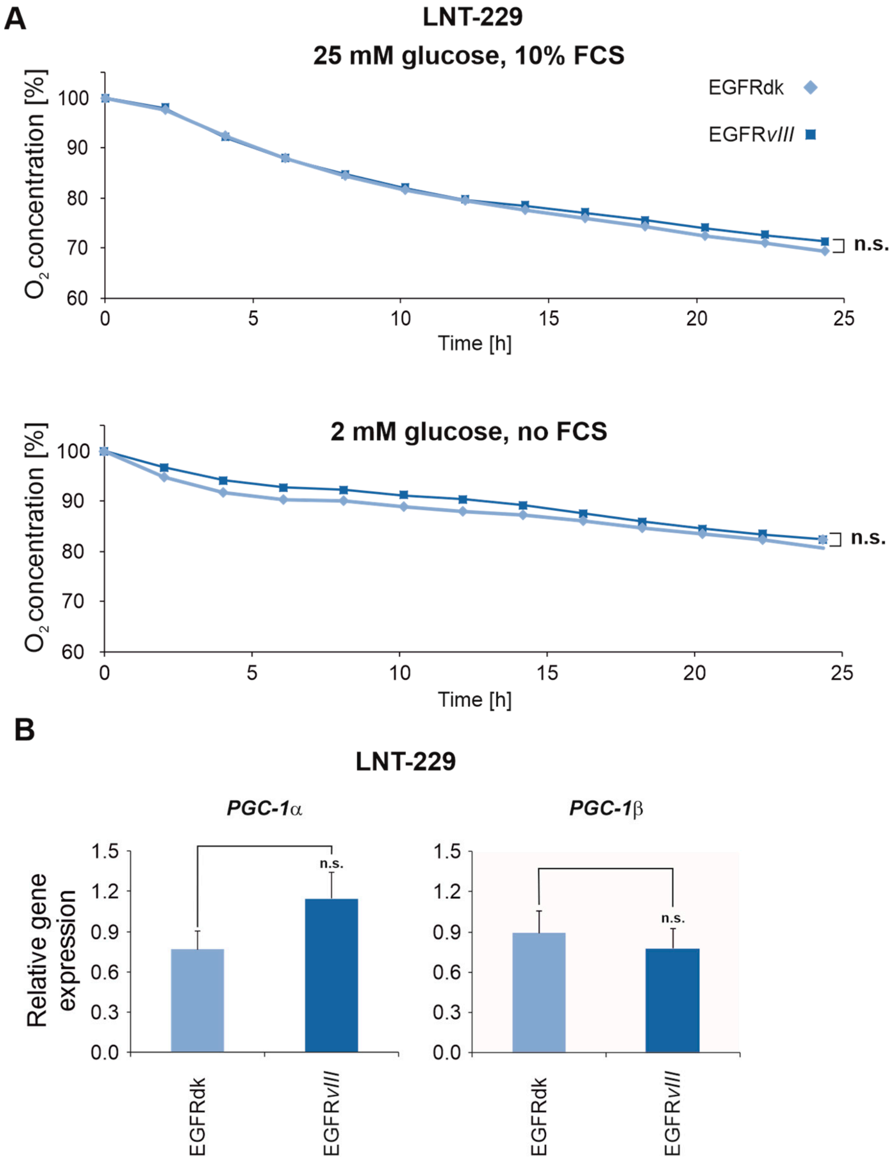

Figure S1. Oxygen consumption and expression of genes of mitochondrial oxidative function are not altered by EGFR*vIII* expression. **A**, LNT-229 EGFRdk and EGFR*vIII* cells were incubated in medium containing 10% FCS with 25 mM glucose or in glucose restricted (2 mM glucose) serum–free medium. Oxygen consumption was measured by a fluorescence-based assay (*n* = 3, mean, n.s. = not significant). **B**, cDNA of LNT-229 EGFRdk and *vIII* cells was generated. Gene expression of *PGC-1α* and *PGC-1β* was quantified by qPCR, values are normalized to *18S* as well as *SDHA* housekeeping gene expression (*n* = 3, mean ± S.D., n.s. = not significant).

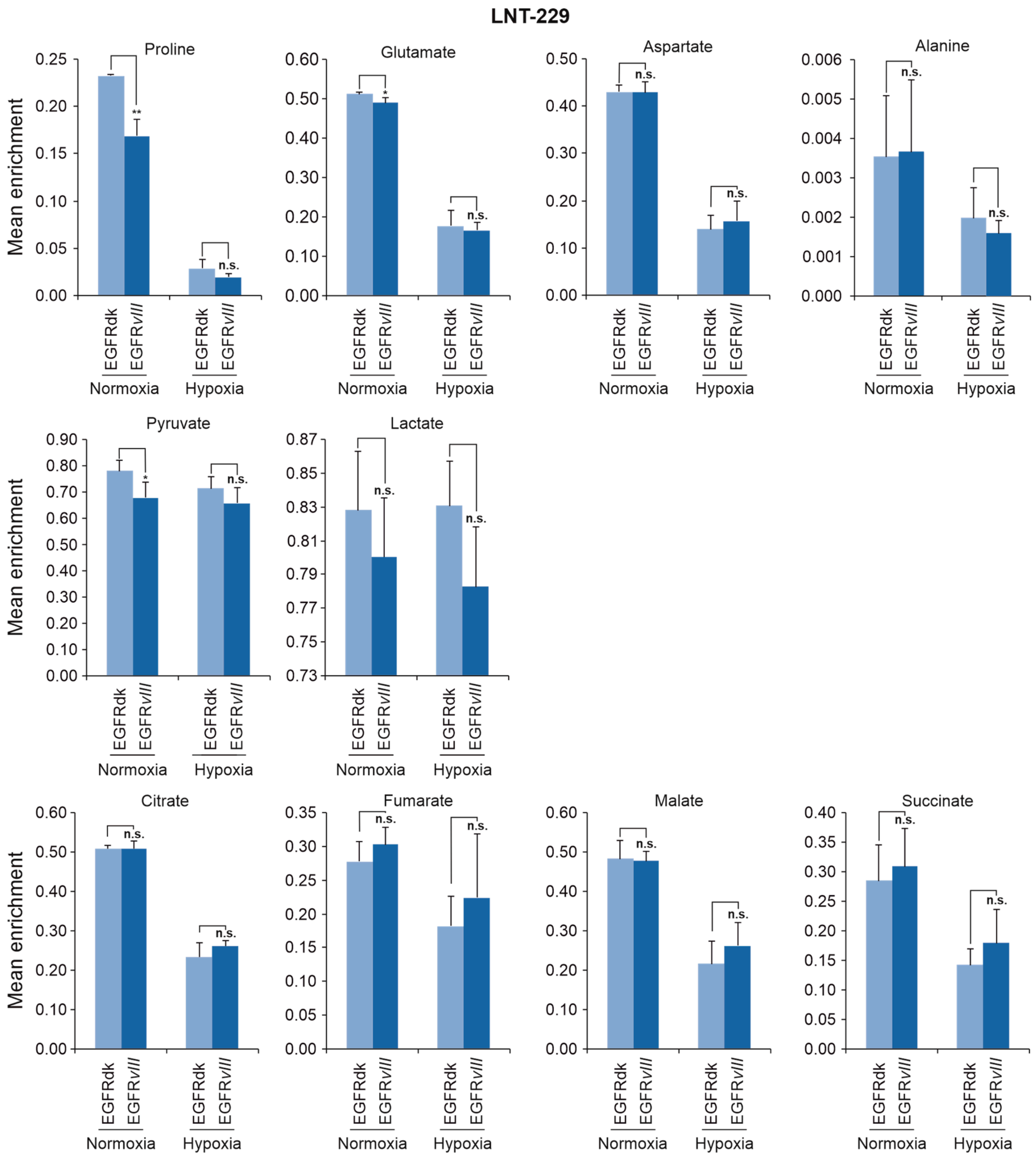

Figure S2. Intracellular metabolic flux of glucose in LNT-229 EGFRdk and EGFR*vIII* cells measured via ^{13}C-flux-analysis. Cells were exposed to glucose restricted (2 mM D-Glucose-[13]C6) serum-free medium under normoxic conditions or 0.1% oxygen. Analysis of amino acid isotopologues was performed by HPLC-ESI-MS/MS. Analysis of isotopologues of organic acids from glycolysis and TCA cycle was performed by GC-MS. (n = 3, mean ± S.D., n.s. = not significant, * $p < 0.05$, ** $p < 0.01$).

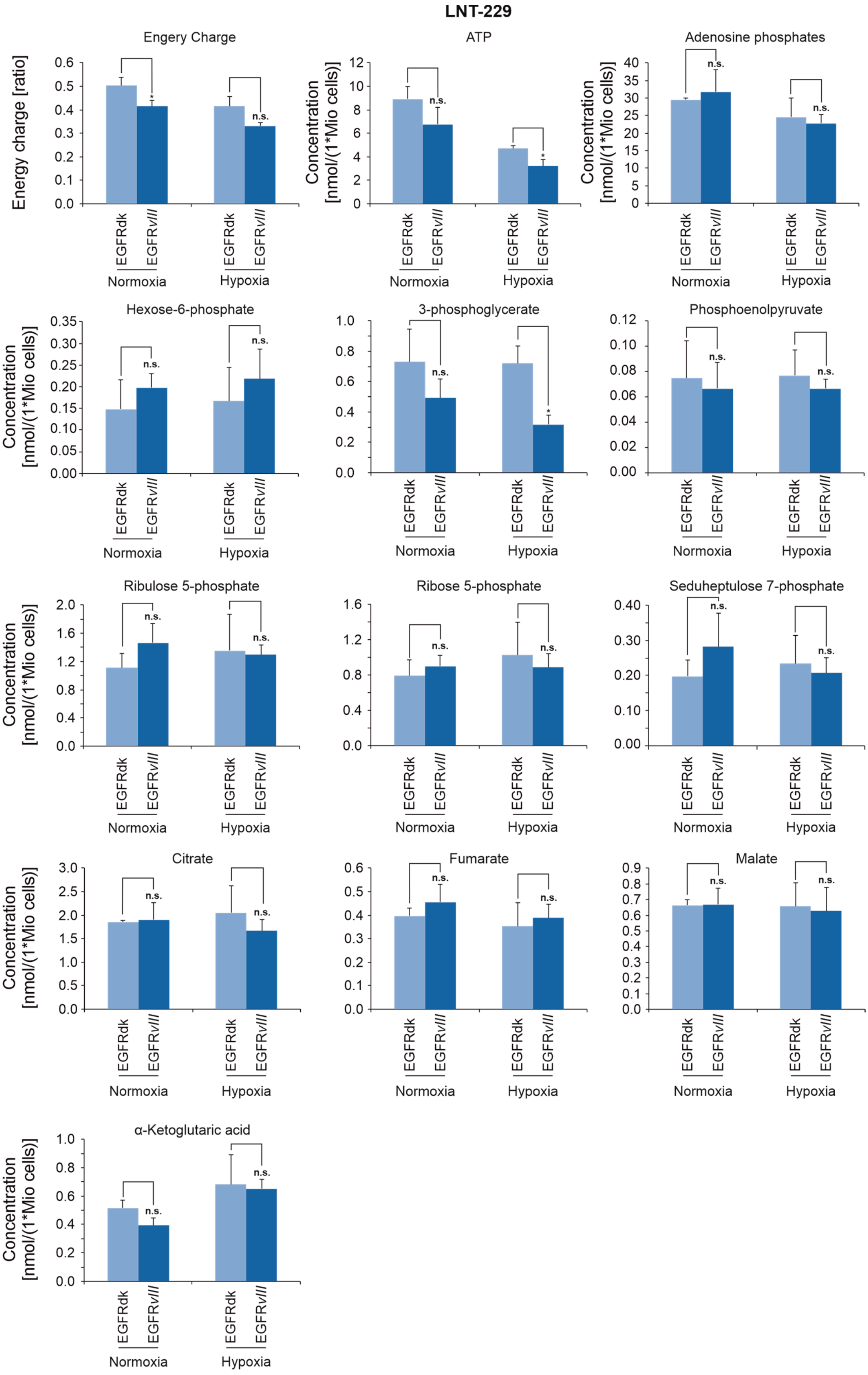

Figure S3. Intracellular metabolic profiles of LNT-229 EGFR*vIII* and dk cells. LNT-229 EGFRdk and EGFR*vIII* cells were exposed to glucose restricted (2 mM glucose) serum-free medium under normoxic conditions or 0.1% oxygen. Intracellular metabolites were analyzed either by LC-MS-MS or GC-MS analysis ($n = 3$, mean ± S.D., n.s. = not significant, * $p < 0.05$).

	TSC2sh	EGFR*vIII*
Aerobic glycolysis	↓	↑
OXPHOS	↑	↔
Pentose phosphate pathway	↑	↔
Serine synthesis pathway	n.a.	↓

Figure S4. Schematic overview of the metabolic changes in TSCsh and EGFR*vIII* cells. ↑: elevated, ↔: unaltered, ↓: decreased, n.a.: not analyzed.

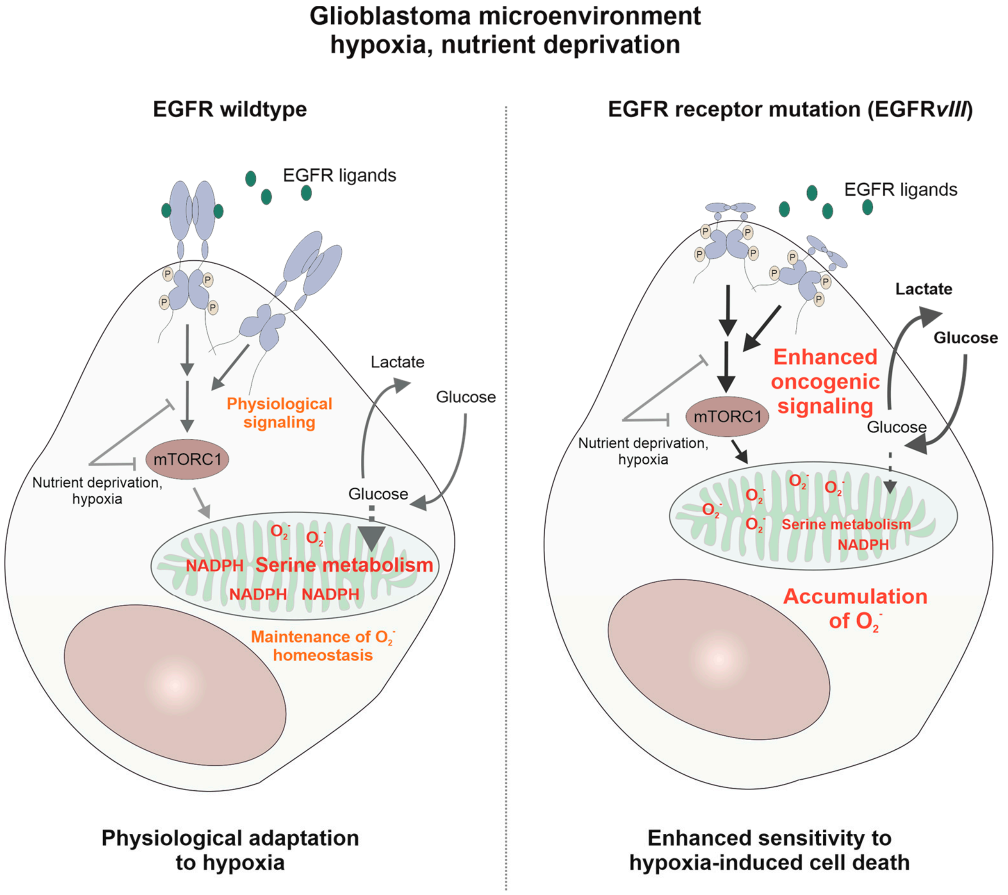

Figure S5. Schematic model of metabolic changes in EGFR wildtype (wt) and EGFR*vIII* cells. EGFRwt cells require ligand binding for induction of signaling which can be inhibited physiologically by starvation and hypoxia. In contrast, EGFR*vIII*-mutated cells display ligand-independent enhanced EGFR signaling which is less sensitive to physiological inhibition. EGFR*vIII* induces enhanced glucose and reduced serine metabolism. As a potential consequence mitochondrial superoxide levels are increased due to a reduction in NADPH culminating in an overall enhanced sensitivity to hypoxia-induced cell death.

Immunoblot of Fig. 1A

LNT-229

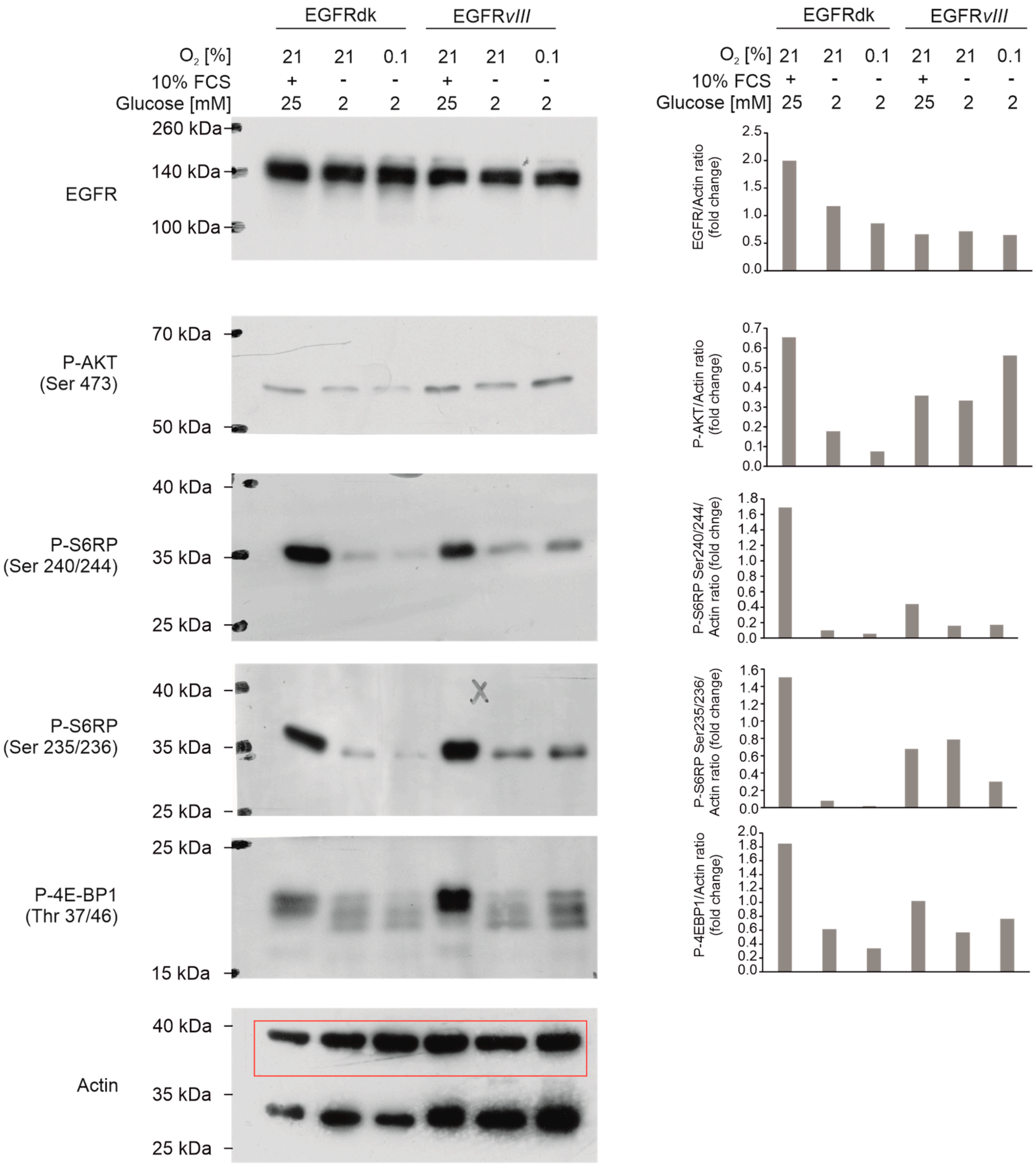

Immunoblot of Fig. 3B
LNT-229 EGFRwt

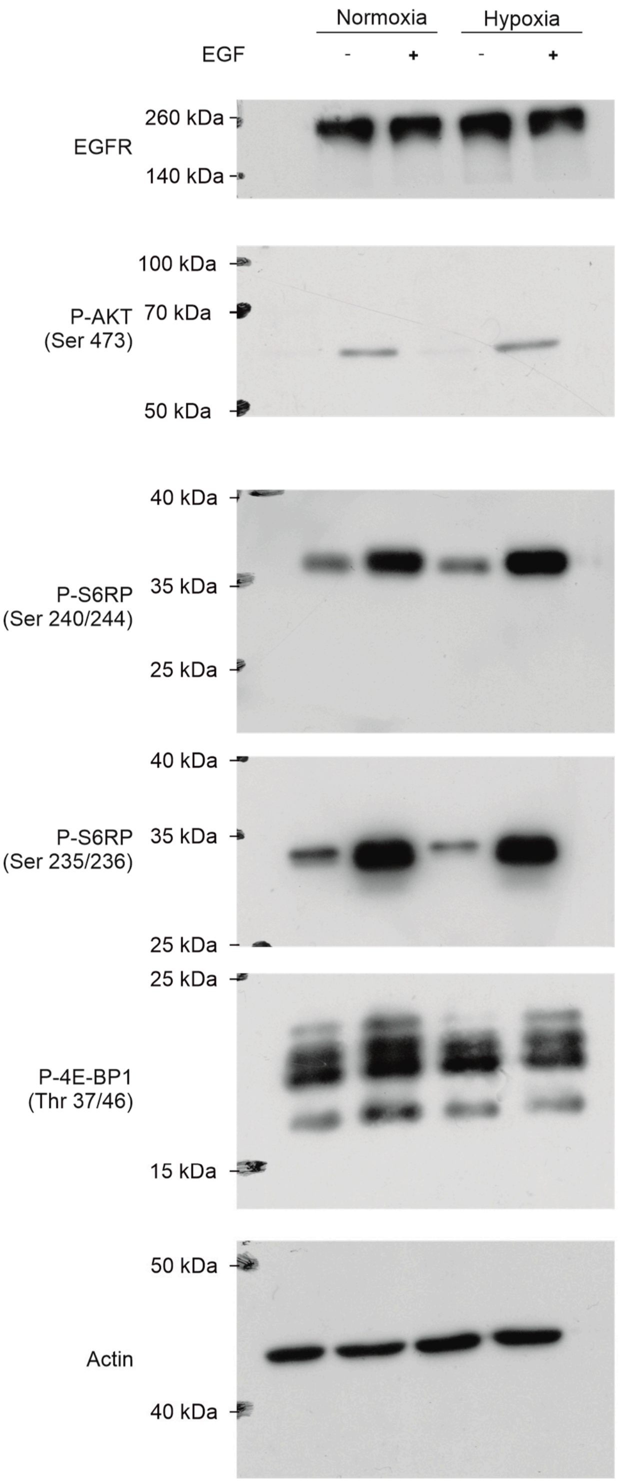

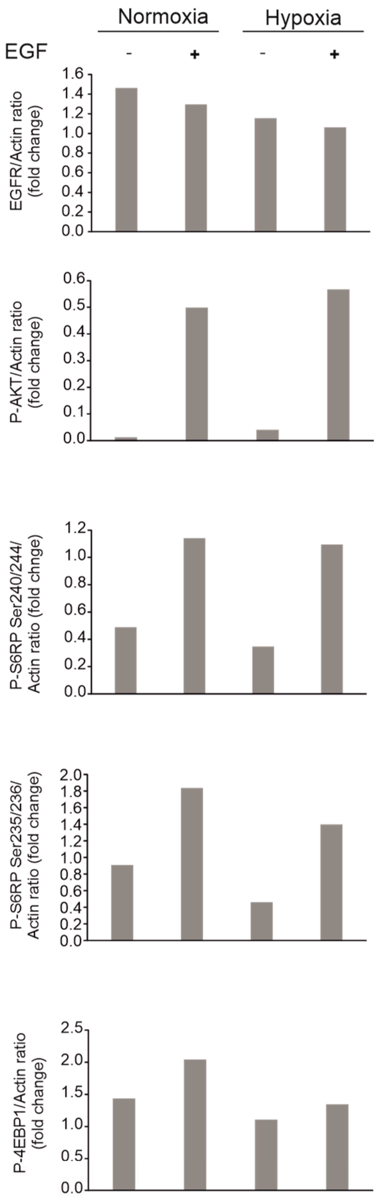

Immunoblot of Fig. 4C

LNT-229 pTet-One EGFR*vIII*, clone 7

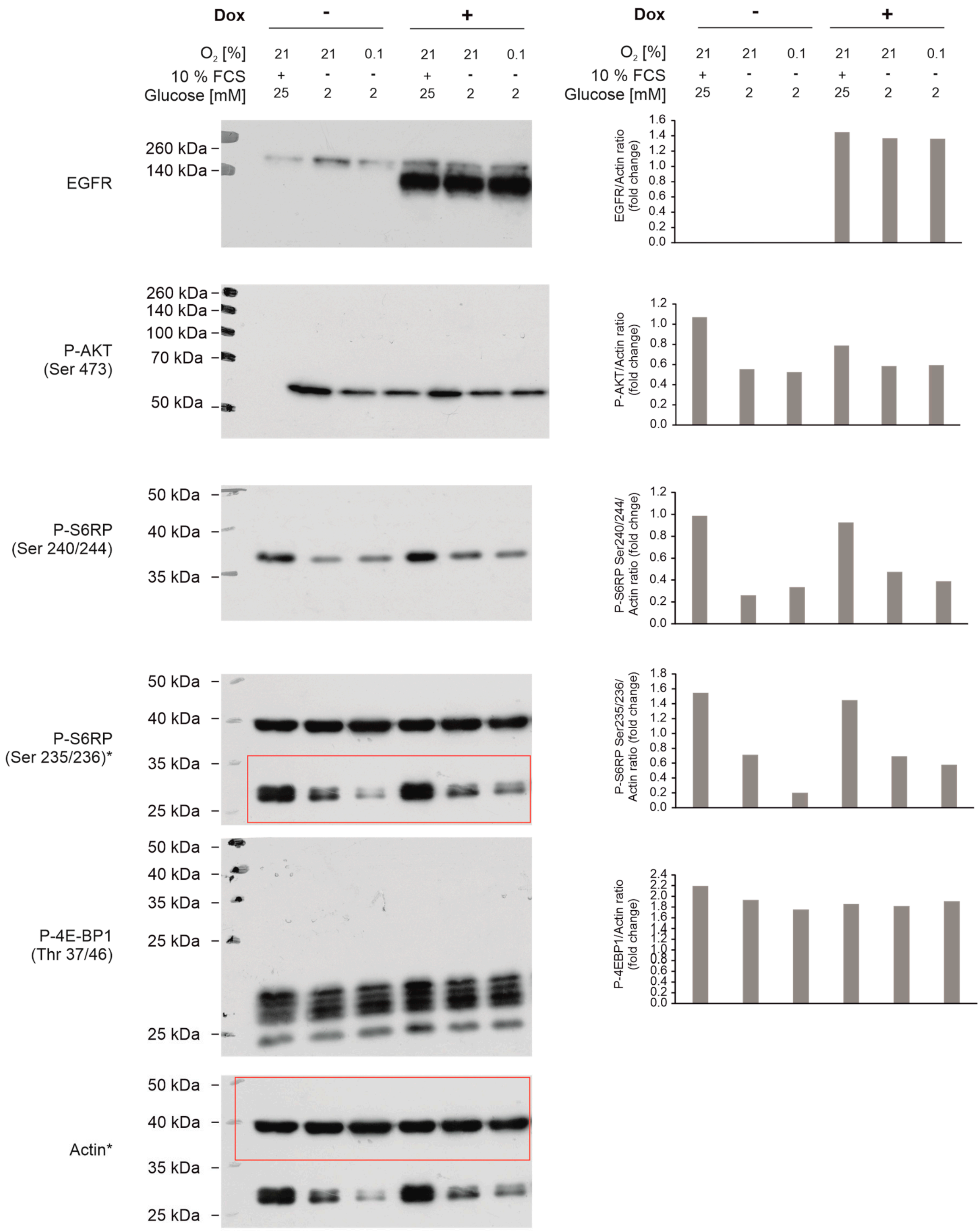

* Please note that P-S6RP (Ser 235/236) and actin were analyzed from the same membrane

Immunoblot of Fig. 4C

U87MG

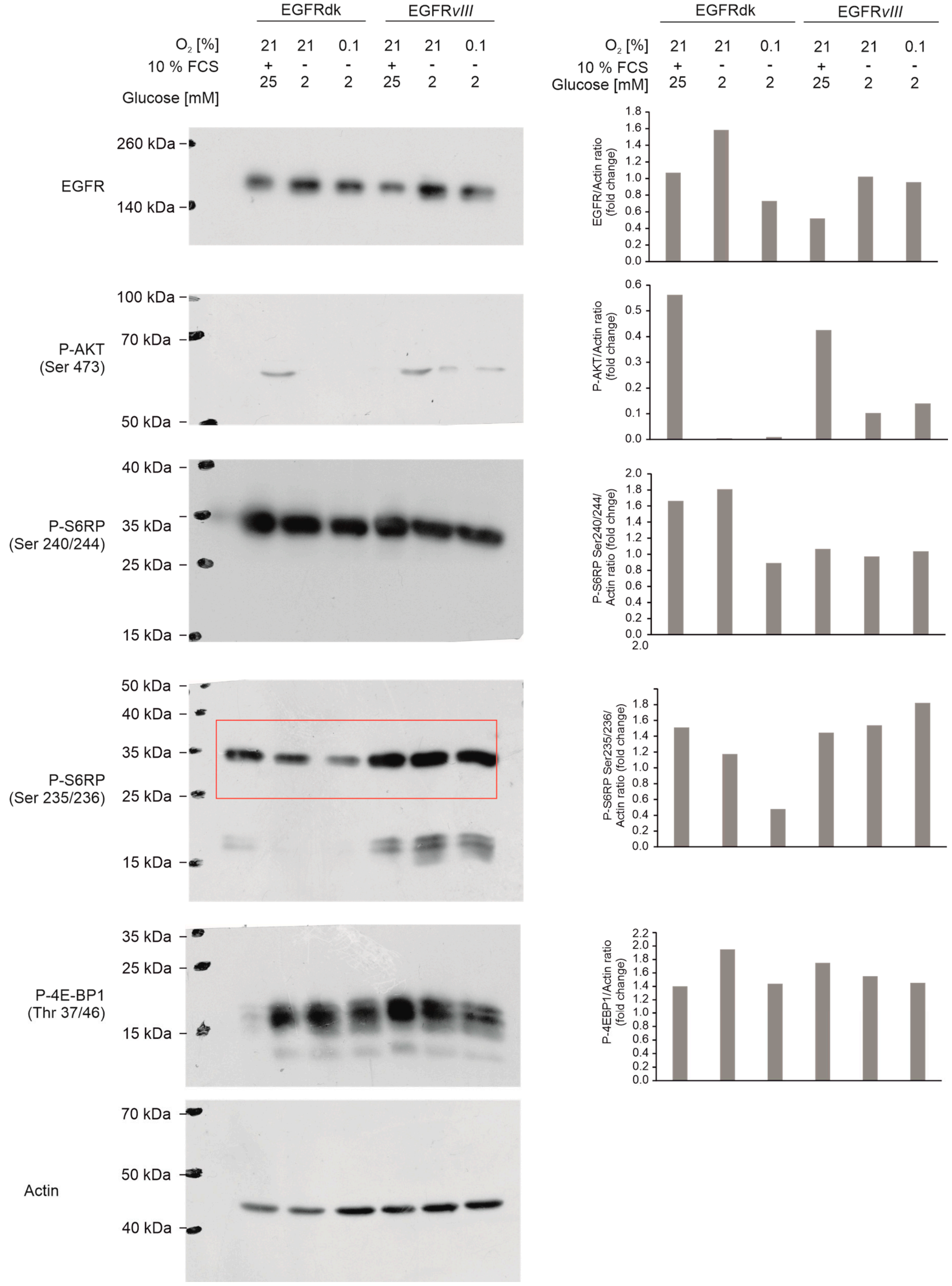

Immunoblot of Fig. 4C

BS153

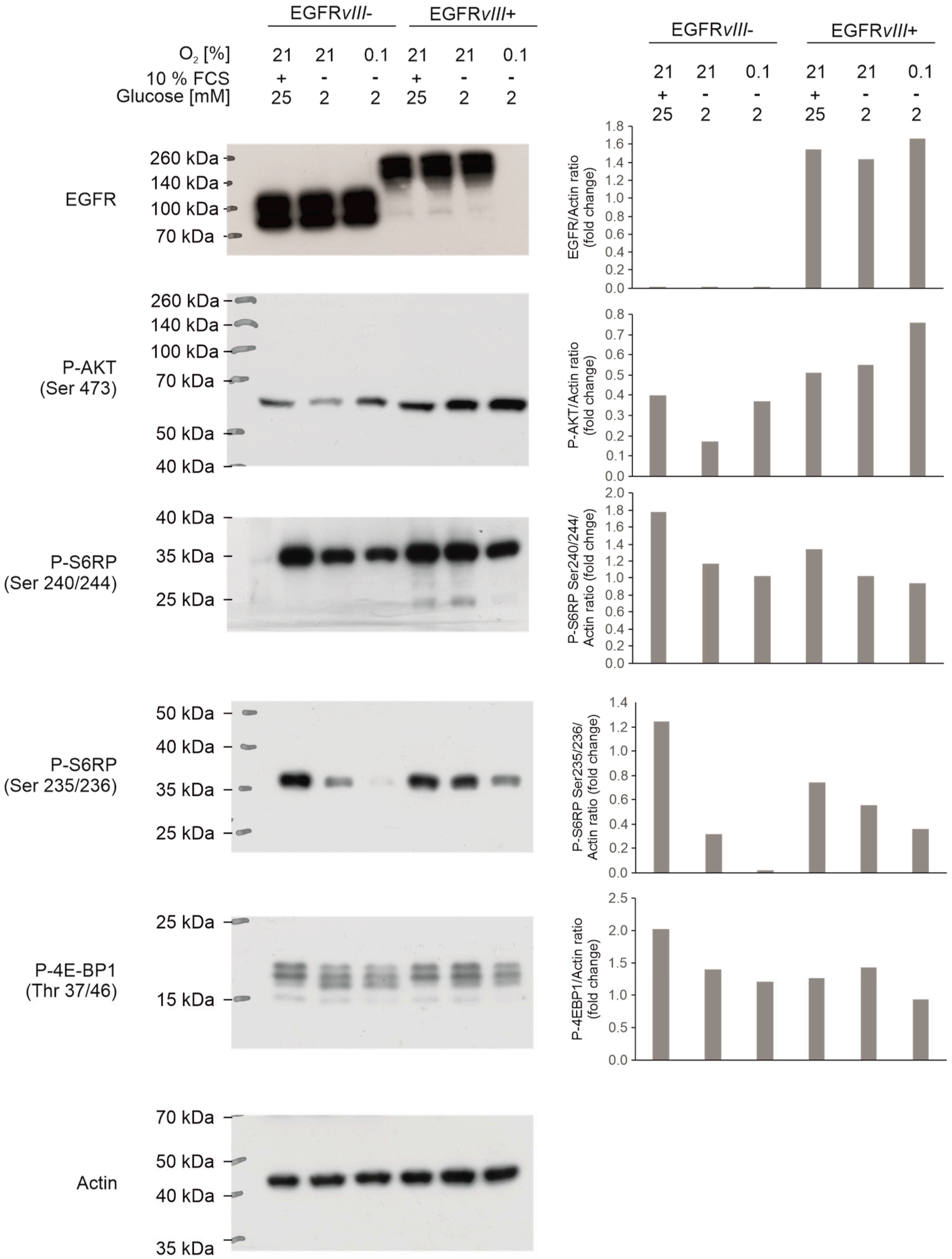

Immunoblot of Fig. 4E

LNT-229

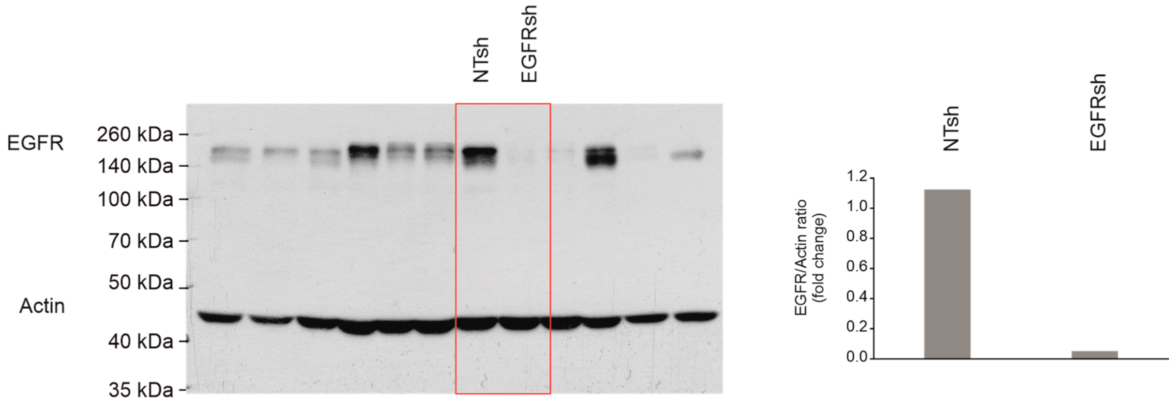

Figure S6: Densitometry readings of immunoblots. Quantification of immunoblot bands was performed by measuring the pixel density of scanned films using ImageJ software (NIH). The ratio to the housekeeping gene actin is shown.

Supplementary Materials and Methods

Table S1. Primer pairs for qRT-PCR analysis. *18S* and *SDHA* were both used as housekeeping genes for normalization.

Gene	Fwd	Rev
18S	5′-CGGCTACCACATCCAAGGAA-3′	5′-GCTGGAATTACCGCGGCT-3′
SDHA	5′-TGGGAACAAGAGGGCATCTG-3′	5′-CCACCACTGCATCAAATTCATG-3′
EGFR	5′-GCGTTCGGCACGGTGTATAA-3′	5′- GGCTTTCGGAGATGTTGCTTC-3′
PHGDH	5′-CTGCGGAAAGTGCTCATCAGT-3′	5′-TGGCAGAGCGAACAATAAGGC-3′
SHMT2	5′-CCCTTCTGCAACCTCACGAC-3′	5′-TGAGCTTATAGGGCATAGACTCG-3′

Stable Isotope Tracer Analysis

For stable isotope tracer analysis, cells were treated with ^{13}C-labelled D-glucose (U-^{13}C6, 99%) for 8 hours, before they were washed three times with phosphate-buffered saline (PBS), harvested in 80% methanol and stored at −80 °C until further analysis. For analysis, the 80% methanol suspension was vortexed and centrifuged (9560 *g*, 5 min, 4 °C). The supernatant was collected and the protein pellet was washed twice with 200 µL 80% methanol and the last wash was centrifuged at 13800 *g*. All extracts were combined and dried in a vacuum evaporator (CombiDancer, Hettich AG, Bach, Switzerland). Samples were re-dissolved in 100 µL water and aliquots were used to measure organic acids and amino acids. Analysis of amino acid isotopologues was performed by HPLC-ESI-MS/MS as previously described [1].The method was modified to monitor an MRM transition for each isotopologue. Analysis of isotopologues of organic acids from glycolysis and TCA cycle was performed by GC-MS. A sample aliquot was dried (CombiDancer Hettich AG) and subjected to methoximation and silylation using the derivatization protocol and instrumental setup previously described [2]. Raw data were corrected for natural isotope abundance and tracer impurity using the IsoCorrectoR package [3].

Quantification of Intracellular Metabolites

Quantification of intracellular metabolites was performed as described[4]. The sampling and processing of the samples were done as previously described [30]. Quantification of metabolites was performed by either LC-MS-MS or GC-MS as described [4–6].

References

1. van der Goot, A.T.; Zhu, W.; Vázquez-Manrique, R.P.; Seinstra, R.I.; Dettmer, K.; Michels, H.; Farina, F.; Krijnen, J.; Melki, R.; Buijsman, R.C.; et al. Delaying aging and the aging-associated decline in protein homeostasis by inhibition of tryptophan degradation. *Proc. Natl. Acad. Sci. U. S. A.* **2012**, *109*, 14912–14917, doi:10.1073/pnas.1203083109.
2. Dettmer, K.; Nürnberger, N.; Kaspar, H.; Gruber, M.A.; Almstetter, M.F.; Oefner, P.J. Metabolite extraction from adherently growing mammalian cells for metabolomics studies: optimization of harvesting and extraction protocols. *Anal. Bioanal. Chem.* **2011**, *399*, 1127–1139, doi:10.1007/s00216-010-4425-x.
3. Heinrich, P.; Kohler, C.; Ellmann, L.; Kuerner, P.; Spang, R.; Oefner, P.J.; Dettmer, K. Correcting for natural isotope abundance and tracer impurity in MS-, MS/MS- and high-resolution-multiple-tracer-data from stable isotope labeling experiments with IsoCorrectoR. *Sci. Rep.* **2018**, *8*, 17910, doi:10.1038/s41598-018-36293-4.
4. Thiepold, A.-L.; Lorenz, N.I.; Foltyn, M.; Engel, A.L.; Divé, I.; Urban, H.; Heller, S.; Bruns, I.; Hofmann, U.; Dröse, S.; et al. Mammalian target of rapamycin complex 1 activation sensitizes human glioma cells to hypoxia-induced cell death. *Brain* **2017**, *140*, 2623–2638, doi:10.1093/brain/awx196.
5. Maier, K.; Hofmann, U.; Bauer, A.; Niebel, A.; Vacun, G.; Reuss, M.; Mauch, K. Quantification of statin effects on hepatic cholesterol synthesis by transient (13)C-flux analysis. *Metab. Eng.* **2009**, *11*, 292–309, doi:10.1016/j.ymben.2009.06.001.
6. Hofmann, U.; Maier, K.; Niebel, A.; Vacun, G.; Reuss, M.; Mauch, K. Identification of metabolic fluxes in hepatic cells from transient 13C-labeling experiments: Part I. Experimental observations. *Biotechnol. Bioeng.* **2008**, *100*, 344–354, doi:10.1002/bit.21747

© 2020 by the authors. Licensee MDPI, Basel, Switzerland. This article is an open access article distributed under the terms and conditions of the Creative Commons Attribution (CC BY) license (http://creativecommons.org/licenses/by/4.0/).
